# Shooting Stars

**Published:** 1833

**Authors:** 


### Art. VII. Shooting Stars.

*An Account of a remarkable exhibition of Shooting Stars, seen at various and distant places, on the night of Wednesday, 13th of November, 1833. Collected and arranged by the Editor.*

As our journal is devoted to the history of every natural phenomenon, which may be *supposed* to be connected with the causes of disease, we propose to record the principal facts, relative to the late brilliant and unexampled display of shooting stars.

In all ages, Epidemic diseases have been suspected, by the people at large, and by the more imaginative members of the profession, to be in some way or other connected with great operations in the earth or the heavens—such as volcanic eruptions, earthquakes, comets, shooting stars, and other phenomena, the causes and effects of which are obscure, or not reducible to any of the *known* laws which govern physical operations. In the year 1800, our learned countryman, Mr. Noah Webster, travelling back to the Noachin Deluge, presented the profession with two volumes of book authorities, to show that great epidemics, are always preceded, accompanied or followed, by striking events in the globe, its atmosphere, or the ether, which stretches out beyond, and in which the planets revolve. The occurrence of the late phenomenon, so soon after the prevalence of the Epidemic Cholera, will undoubtedly be regarded by the Webster school, as affording support to their favourite theory; and, as it is impossible to *disprove* this assumption, we shall admit the propriety; recording that which they will regard as an important item in the history of these epidemic times; and shall therefore proceed to collect from the public journals, and arrange into a narrative, the principal facts relative to that splendid exhibition.

All the world are familiar with the phenomenon called a shooting-star, seen in every country almost every fair night; often in considerable numbers, but in succession. It is obvious the late exhibition was nothing more than the same phenomenon multiplied to an indefinite number, and presenting in a single night, all the varieties of appearance that the solitary shooting-star offers in a succession of centuries.

We had not the good fortune to witness this sublime and beautiful spectacle, and in becoming one of its historians, must transcribe from others. We shall make these transcripts from such a number of the publications in the gazettes of the country, as will afford a permanent record of the aspect which the heavens presented over various and distant places, beginning with Cincinnati. The phenomenon seems to have commenced, at this place, before midnight; and gradually attained its maximum of brilliancy from 3 to 5 o'clock, though it evidently had not ceased at daylight. A writer in the Cincinnati Gazette gives the following statement of his own observations:

"Yesterday morning, about a quarter of 4 o'clock, there was a brilliant exhibition in the heavens. The whole firmament was incessantly traversed, in all directions, by luminous streams of electric light. They all appeared to proceed towards the earth, commencing at various elevations; from about 30 degrees from the horizon to the zenith. The streaks of light were of different intensity, sometimes contracting into a slender line, at others presenting the appearance of a broad rectilineal flash. Frequently when approaching the horizon, they exhibited, at their termination, an appearance somewhat similar to an explosion, leaving, for some seconds, a rich mellow light, blended with all the hues of the rainbow. They moved with various velocity—sometimes rapid, at other times slow in their progress. In their course they ordinarily resembled shooting-stars. The entire vault exhibited the scene of a magnificent pyrotechnic display."

At Springfield, in this State, the editor of the Pioneer observed that—

"The meteors were mostly of the usual size, but at times a larger and more glaring ball would start forth, leaving a broad, bright flame on its track, and shedding its light over all. Their course held generally, and we believe without exception, towards west by north. We could detect no change in the appearance of the atmosphere. The morning was mild and clear. The shooting of these lights prevailed north of this, but not, as in the south, at the extreme verge of the horizon. We "took the field" of observation at about four o'clock, and continued on the *qui vive* until the "greater light" put out these lesser lights. We understand that this phenomenon was to be seen as early as midnight. If it prevailed more at one time than at another, during the two hours that we observed it, it was at five o'clock."

A writer, who is a physician, gives in the Somerset, Ohio Post, the following account:

"The splendid meteoric phenomenon of the morning of the 13th inst. having attracted considerable attention, some account of it, and a few words on meteors in general, may perhaps be entertaining. I did not observe it much before 5 o'clock, but as the coruscations were said to be then as brilliant as they had previously been, by those who had witnessed them for upwards of an hour, a description of them from that time may perhaps suffice for the whole phenomenon. The heavens were then lighted up by numerous scintillations streaming in every part, and apparently in every direction; but upon more attentive observation, I perceived they all flowed in the directions of radii from a common centre, which centre was about twenty-five degrees south-east of the zenith. They commenced their courses at various distances from it, and appeared to be directed towards the horizon, with the exception of those between their centre of repulsion and the zenith, which appeared to direct their courses upwards; but I imagine that their *apparent* directions were occasioned by their great distances from us, and that they really shot in lines parallel to the earth's surface. They moved with the velocity of what are commonly called falling-stars, and always, as far as I could observe, in right lines, leaving luminous traces behind them. They differed in magnitude and brilliancy; the luminous lines left by the smaller ones, faded quickly, whilst those which were large, cast a strong light upon the objects around us, and left streaks which continued for several minutes, perhaps nearly as much as ten in some instances, which streaks gradually became bent in various shapes, as if acted upon by wind. and faded away into nebulous patches, like faintly luminous clouds. No sound that we could distinguish was emitted by them. They generally became fainter and less numerous as day broke; probably by the superior light eclipsing them. There was a slight frost at the time, with a light air stirring from the west, from which quarter a stronger wind with rain had proceeded for two or three days before. The heavens were cloudless, and continued so the greater part of the ensuing day."

An observer at Warren, in the eastern end of this State, gives the following account of them, as seen at that place:

"They resembled somewhat, the rockets discharged in displays of artificial fire-works, or more, the shooting-stars that are often seen darting through the air at evening. In size they varied from that of the planet Venus, which was then ten or fifteen degrees above the horizon, to a mere luminous point; and they invariably left behind them a luminous track, visible for several seconds, according to their magnitude. The largest emitted, during their progress, coruscations that appeared like flashes of lightning. It is impossible to give any definite idea of the number visible at any one moment: they varied at different times. One person compared them to large flakes of snow at the close of a snow storm—they were not, however, as numerous.

At times they fell in showers, and were much thicker in some directions than in others;—then again they would appear equally numerous in every part of the skies, and at no time, while it was dark, could the eye be fixed on any one point for a moment, without seeing a considerable number. They appeared to form high in the atmosphere, and dart towards the earth with about the same rapidity that a rocket shoots into the air, and finally become extinct within from fifty to one hundred feet of the earth, leaving, for a time, as before mentioned, a luminous trail behind them. Their direction was invariably from the south-east to the north-west, from high in the firmament toward the earth, forming a very acute angle with a line perpendicular to the horizon. The whole time they were evident was about three hours. The driver of the Pittsburgh stage informed me, that he left Youngstown at 3 o'clock, that they were not then to be seen, but that he first observed them when a mile or two north of that town, and that they were constantly visible until he arrived in this place. The distance between the two towns is about twelve miles.

I was called up at 5 o'clock, and watched them attentively until 6, when they were rendered invisible by the diffusion of daylight. Occasionally one could be seen so long as the planet Venus was discernible to the naked eye. Before the day dawned, the stars were unusually brilliant; the atmosphere was clear and unobscured by clouds, except a thick, dark body of haze, lay just above the horizon, several degrees below the quarter from which the meteors appeared to proceed. A slight breeze was blowing from the same point of the compass during the whole time.

Soon after I was awakened, my attention was attracted to a luminous spot in the north-east, which had a strong resemblance to a new moon, slightly obscured with clouds, when two or three hours high. It continued visible about fifteen or twenty minutes, and gradually disappeared. I am credibly informed that at 4 o'clock it was very brilliant, in the form of a pruning-hook, or an italic letter *S*, and appeared to be fifteen or twenty feet long.

The weather through most of the preceding day was mild, damp, and somewhat cloudy; at evening it became clear, and so cold that during the night the mud in the streets froze sufficiently hard to bear a person. At ten o'clock a hazy cloud lay in the north, and was bordered on its upper edge with the aurora borealis. A few minutes afterwards, on retiring to my chamber, I observed my clothes to be fully charged with electricity, which was, upon any considerable motion, emitted in brilliant sparks."

At Buffalo, New York, the following account was published:

"Very interesting atmospherical phenomena were visible, a little before daylight this morning, in this city. My attention was first called to them about half past four, A. M. The wind, which had been very heavy for many hours, had abated considerably, every vestige of clouds had disappeared, and the stars were shining through an unusually clear atmosphere. The air from within 20 feet of the earth to apparently the ordinary height of the clouds, (half a mile,) was filled with phosphoric particles, which were continually and successively becoming luminous. They all passed, while visible, with great velocity through the air, but in no uniform direction; some rose, some fell, others moved horizontally, and others, again, at every conceivable angle, to these several courses. Their colour was a vivid white, resembling that of the flame of steel wire burning in oxygen. Their sizes were various, but mostly about the apparent size of stars of the second magnitude. The distances of their luminous flights were various and not easily calculated. Many of them, in their passage, left a luminous line in the air, of their own colour, which was often several yards in length, and usually remained visible from one to three seconds. I have said there was no cloud: there was none, but the whole atmosphere was constantly, at very short intervals, illuminated by flashes of light, in no way differing from ordinary silent electrical explosions. The Aurora Borealis, during the whole time of my observations, which was about half an hour, was distinctly visible, though by no means so brilliant or so active as that meteor usually is, when visible here.

Upon the whole, the scene was by far the most sublime meteoric display I ever beheld; and I only regret that the unfavorable hour of its occurrence deprived me of an opportunity of observing the influence that such a singular electrical state of the atmosphere may be supposed to have exerted upon the magnetic needle."

At Connersville, Indiana, they were observed, and described in the following terms, by a writer in the Port-Folio:

"The evening referred to was cold and clear. Not a cloud was discoverable above the horizon. About 3 o'clock in the morning the atmosphere was literally filled and illuminated with meteors, resembling what are called falling-stars. They moved apparently from the zenith, to every point of the compass. In their descent they reflected a very brilliant light, which was nevertheless whiter than the light reflected by a common blaze of fire. The *bolides* (fire-balls) or particles, were of different sizes; some apparently of the size of the fixed stars, and others five or six times as large. Those of the largest size, on their descent, left behind them a luminous path, which was perceivable 5 or 6 seconds after they had ceased to exist. In the space of 30 minutes after their first appearance, the scene was truly grand and terrific. The atmosphere presented something like the same appearance as when globules of ice or hail, are falling in large quantities, when the sun is unclouded. But the contrast which was created by the darkness of night, made the scene imposing beyond the power of description. Accompanying these appearances was not the slightest noise. The stillness of night was not disturbed by the softest zephyr. The meteors continued to float, though gradually abating in quantity, until the rising sun closed the scene."

The editor of the Lexington Ky. Intelligencer, has given the following notice of this phenomenon:

"The whole firmament was filled by thousands if not millions of shooting and blazing meteors, traversing each other's track at all angles, horizontally, longitudinally, and in circles, and approaching the earth so nearly as to illuminate the streets, and to resemble an incessant shower of flaming comets. The appearance of the meteors varied in size, from the circumference of a man's head to that of a dollar, and after explosion, assumed every imaginable singularity of form. This natural display of fire-works continued from 2 until 5 o'clock in the morning. The sky was perfectly clear; the atmosphere damp, occasioned by the fall of rain on Monday, and the temperature was about 60 of Fahrenheit, where it had ranged for several days preceding."

Without multiplying the descriptions of this phenomenon, as seen in the valley of the Mississippi, we may state that, it was observed as far north as Detroit, south as New-Orleans, and west as St. Louis.

Of the appearance which it exhibited on the eastern side of the mountains, the following notices may suffice. At Augusta, in Georgia, it was described in these words:

"For several hours last night the heavens presented an appearance seldom if ever witnessed before. Hundreds and thousands of meteors, resembling those occasionally seen in our cloudless nights, and of different degrees of brilliancy, were seen in all directions, darting through the sky. Some appeared no larger than a small spark, and to the eye seemed to move but a short distance; others were exceedingly brilliant, and pursued their burning way over one fourth or one third part of the visible expanse. A few shed abroad a sudden gleam as bright as lightning in a dark night, or equal, perhaps, to the light of the full-orbed moon, and left behind them a luminous pathway, which would continue twenty or thirty seconds. The attention of the writer of this notice was directed to the strange phenomenon a few minutes after 5 o'clock this morning, (or just at the break of day,) though it was seen by others in our town as early perhaps as 2 or 3 o'clock. By examining the meteors from different apartments of my house, I soon perceived that they all moved off in diverging directions, like the rays of a lamp, or the radii of a circle. This induced me to take my position in the open street, where I could survey the wonder more accurately. I discovered that all parts of the heavens were ornamented in rapid succession by these darting gems. Multitudes might occasionally be seen at the same instant. They commenced their career at all points of elevation above the horizon—and it was easy to perceive that, in all cases, the lines of their direction, if continued upward, would have met at the zenith. Indeed, many of these blazing travellers commenced their course near this focus, and hurried off to every point of the compass. Of the hundreds that I noticed, not one crossed the focus, not one intersected the path of a fellow-traveller, or of any that had gone before. The radiating appearance of the meteors was probably an optical illusion, as bodies falling from an immense height seem to recede from us as they approach the earth. The whole scene was indescribably beautiful; to some, perhaps awful, because so new and unexpected. Many in our city were at first much alarmed: it seemed that the stars of heaven were falling from their places.

The interesting scene lingered upon our eyes until the brighter glories of the morning shrouded it from our view. A few of the brightest meteors, however, were seen maintaining their contest with the morn, till as late as ten or fifteen minutes past 6. Here is work for philosophers. We cannot suppose that this singular phenomenon affords any evidence that the common laws of nature have been transgressed; but nature has never before appeared before our eyes in such garments, and perhaps seldom to the eyes of others. Such scenes will be regarded by all pious minds as furnishing occasions for holy wonder and devout adoration."

The Charleston S. C. Courier contains the annexed notice:

"The atmosphere in and about the city, was, on Tuesday night last, illuminated with a brilliant and extraordinary meteoric display. It consisted of myriads of falling or shooting-stars, some of a very large size, darting in an oblique direction towards the earth from every part of the heavens, and occasionally exploding like rockets. The luminous appearance commenced about midnight, were most brilliant between 3 and 4 o'clock A. M., being assimilated by those who witnessed them to a fiery rain, or hail, and continued until near sunrise. We understand that a very large meteor exploded immediately over the City Hall. A sudden change of temperature, from hot to cold, which took place during the night, was probably closely connected with the origin of the phenomenon. We have been informed by Capt. Jackson, of the Revenue Cutter Jackson, who was at sea that night, at the distance of 9 miles from land, that the heavens were illuminated with the meteors during nearly the whole night, as far as the eye could reach, in every direction, presenting a spectacle of uncommon magnificence and sublimity, attended with frequent explosions resembling the discharge of small arms. We learn also that a meteor of extraordinary size was observed at sea to course the heavens for a great length of time, and then explode with the noise of a cannon."

Mr. F. G. Smith made the following observations on this phenomenon, at Lynchburg, Va.:

"On this morning (Nov. 13) between 2 o'clock and daybreak, we were presented with a most beautiful display of electrical excitement in the upper regions of the atmosphere, probably not excelled in interest by the similar meteoric phenomenon of Nov. 1802.

At 10 o'clock last night, I was struck with the uncommon transparency of the atmosphere and brilliancy of the stars. Soon after having my attention thus called to the peculiar state of the air, I felt a slight repetition of the tremulous motion of the earth, which has repeatedly been observed in this vicinity of late.

The shooting-stars, of which we had so impressive an exhibition this morning, made their first appearance in our hemisphere between 2 and 3 o'clock, but I did not notice them until about 5 o'clock. From the vast number and brightness of the meteors, the sight was, at that time, indescribably beautiful. Their general course was from the south-east to the north-west, the most of them appearing to the south-west of our zenith. They first came into view 20 or 30 degrees to the east of our celestial meridian, and extended their flight 40 or 60 degrees to the west of it. Their general motion was probably horizontal, although, from the position of the observer, they seemed to fall. Their path was marked by a train of light which was most brilliant near the point of their disappearance, continuing from 3 to 7 or 8 seconds, and sprinkling the heavens with long bright dashes of light, resembling in their form the marks made on the window by the first drops of a shower driven against the glass. The colour of the light was generally a pure white, but sometimes tinged with a reddish hue; and so great was the number and frequency of the meteors, as to illuminate the night sensibly, though slightly. The average flight of each ball was over an arc of about 50 degrees. The phenomenon was most brilliant to the south and west of Lynchburg, at an elevation of from 30 to 69 degrees. The meteors vanished from sight without a visible or audible explosion, and for the most part without scintillations.

No appearance of the Aurora Borealis was observed, nor the slightest vapor of any kind. The air continued, as on the evening before, entirely pellucid.

At half past 6 o'clock the thermometer stood at 51 degrees, Fahrenheit; the barometer at 29 inches and four tenths, and the hydrometer at about 28 degrees. No change was noticeable in the magnetic dip, variation or intensity. Gold-beat electrometers were excited by a touch; Bennet's placed on the prime conductor, with the cushion insulated, rose on a slight motion of the machine. The pendulum of D. Luc's *dry pile* was accelerated."

The following is one of the accounts of this phenomenon as seen at the city of Washington:

"This morning, about half past four o'clock, our attention was arrested by something which appeared like what is called a falling-star; pretty soon another, and another appeared, so as to produce the impression that they might be bright sparks from a neighboring chimney. Their number and magnitude increased gradually, till, upon going out into the open air, they presented one of the most extraordinary and sublime spectacles which we have ever witnessed. They appeared to shoot, generally, from a point a little south-east of the zenith, diverging, at various angles, in all directions, keeping up a continual shower—though they appeared also to move by fits, with intervals about such as usually occur between the flashes of a sheet of lightning; some of them being so brilliant as to cause a general illumination of considerable brightness. Many of them left their traces in the air, which hung like swords of fire above the earth, for a minute or more. Ten or fifteen of these aerial weapons were often glancing at once. Some of them became crooked and irregular, before they disappeared. The air was remarkably clear and pure, and the stars shone with unusual brilliancy. This splendid exhibition, after attaining its height of grandeur, gradually died away, with the increase of twilight, till it either ceased or became invisible.

The directions of the shooting fire may be supposed to have been generally parallel; and, in that case, they were at a very small angle from the south-east. By the laws of perspective, their starting distance from the earth must have been equal to their distance when they disappeared, as their last apparent distance from the zenith line was to their first apparant distance from the line. Many of them increased their apparent distance from the zenith line probably, in a tenfold degree. Supposing that their last distance from the earth was five miles, their first distance must have been five times ten, or fifty miles. These measures are, of course, only relative."

A Baltimore observer, Mr. Kenny, gives the subjoined statement:

"My attention was arrested at about 4 o'clock this morning, by an unusual number of meteors which I supposed to be flying horizontally in all directions from one centre which appeared to be directly over the spot where I then stood; but on moving to a distance of about one and a half miles, I found the centre appeared still to be over me, and those meteors which appeared there left a much shorter train than those which appeared in any direction around me, from which I concluded that their course was perpendicular. Their number increased from four until half past five o'clock, when it seemed to rain fire, and while I stood at the corner of Charles and Fayette streets, one very bright trail appeared to me directly over Charles street. At first it was straight, the two ends then curling towards the west, till they formed a neat figure of three, after which the ends uncurled, turning towards the east till they came together and formed a straight line which then spread into the appearance of a light cloud, and disappearod after remaining visible at least ten minutes."

The same gentleman has made another communication on this subject, a part of which we shall likewise extract:

"When I first discovered them, it occurred to me that the atmosphere in the region where they originated, must be very much agitated; to ascertain that fact, and also the nature of the materials of which they were composed, I was led to devote my attention from four till seven o'clock, during which time I traversed some of the streets of Baltimore from north-west to south-east, without being able to come within reach of one of them. Although some few of them took a horizontal direction, yet the great majority of them appeared to descend directly towards the earth, but not one of them reached it within my view. Their commencement was generally very small, but they increased as they proceeded for a time, and then decreased in about the same proportion as they increased, leaving a light blaze, narrow at each end and wide in the middle. Some, after appearing to be nearly exhausted, would rekindle and proceed a second time, and some few a third time, leaving behind them as many links or trails, wide in the middle and narrow at each end, only connected together by a fine thread. Some of those trails would bend into various forms, and some few of the more prominent ones, after they were lighted up and had proceeded some distance, would explode and fly in all directions. The morning being clear, and the stars bright, furnished an opportunity to discover that the atmosphere was a perfect calm, as not one of those lights or trails after its own strength was exhausted, was wafted by the wind, but remained in the same spot where it was created till it became extinct. Yet many very brilliant ones were rapid in their motion, and left no trail behind them, but became extinct in an instant. Some of our city watchmen discovered them about two o'clock, from which time their number increased till about half past five o'clock, after which there was a gradual decrease in number until the light of the sun obscured them."

Several New York accounts of these meteors have been published. We shall extract but part of two of them, from the Commercial Advertiser:

"Yesterday's change of weather, pencilled, near sunset upon the western skies, the brilliant hues of a summer's eve. As the curtains of darkness were spread around, the broad belt of the heavens visible in our hemisphere, became studded with the bright gems of night. A strong piercing wind cleared the atmosphere of every offensive vapor, and braced the nerves, at the same time that it gave effulgence to the bright surrounding scene. Somewhat before twelve o'clock, the meteors so often seen on summer evenings, and commonly called shooting-stars, were observed to fall with unusual frequency and splendor. They continued from that hour to flash athwart the skies, more and more, until they were eclipsed by the glories of the rising sun this morning. From four to six o'clock, they were most numerous and refulgent. Within the scope that the eye could contain, more than twenty could be seen at a time, shooting (save upward) in every direction. Not a cloud obscured the broad expanse, and millions of meteors spread their way across it, on every point of the compass.—Were it possible to enumerate them, in the swiftness of their arrowy haste, we might venture to say, that for the two hours intervening between four and six, more than a thousand per minute might have been counted. Their coruscations were bright, gleamy and incessant, and they fell thick as the flakes in the early snows of December. In one instance we distinctly heard the explosion of a meteor, that shot across the north-west, leaving a broad and luminous track; and witnessed another which left a path of light that was clearly discernible for more than ten minutes after the ball, if such it be, had exploded. Its length was gradually shortened—widening in the centre, and apparently consisted of separate and distinct globules of light, drawing around a common centre, glimmering, less and less vividly, until they finally faded in the distance."

"The meteoric shower of the 13th inst, was a rare phenomenon.

At half past four o'clock, A. M., I first observed it, and continued to notice it until its termination, at 6 o'clock A. M.

From a point in the heavens, about fifteen degrees south-easterly from our zeni h, the meteors darted to the horizon in every point of the compass. Their paths were described in curved lines, similar to those of the parallels of longitude on an artificial globe.

They were generally short in their course, resembling much an interrupted line, thus ——. ———. ——————. They ceased to appear when within about ten degrees of the horizon.

I did not see a single meteor pass the meteoric pole which I have described—nor one pass in a horizontal direction.

Several of them afforded as much light as faint lightning. One in the north east was heard to explode with a sound like that of the rush of a distant sky rocket. The time from explosion to the hearing was about twenty seconds—which gives a distance of about five miles.—It left a serpentine cloud of a bright glowing color, which remained visible for about fifteen or twenty minutes.

Millions of these meteors must have been darted in this shower."

The last account we shall extract, is that of Professor Olmstead, of Yale College:

"About daybreak this morning, our sky presented a remarkable exhibition of fire-balls, commonly called shooting-stars. The attention of the writer was first called to the phenomenon about half-past five o'clock, from which time until sunrise, the appearance of these meteors was striking and splendid, beyond any thing of the kind he ever witnessed or heard of.

To form some idea of the phenomenon, the reader may imagine a constant succession of fire-balls, resembling sky rockets, radiating in all directions from a point in the heavens near the zenith, and following the arch of the sky towards the horizon. They proceeded to various distances from the radiating point, leaving after them a vivid streak of light, and usually exploding before they disappeared. The balls were of various sizes, and degrees of splendor; some were mere points, but others were larger and brighter than Jupiter or Venus; and one seen by a credible witness, before the writer was called, was judged to be nearly as large as the moon. The flashes of light, though less intense than lightning, were so bright as to awaken people in their beds. One ball that shot off in a north-west direction, and exploded near the star Capella, left, just behind the place of explosion, a phosphorescent train of peculiar beauty. This line was at first nearly straight, but it shortly began to contract in length, and dilate in breadth, and assume the figure of a serpent folding itself up, until it appeared like a small luminous cloud of vapor. This cloud was borne eastward by the wind, opposite to the direction in which the meteor had proceeded, remaining in sight several minutes. The light was usually white, but was occasionally prismatic, with a predominance of blue.

A little before six o'clock, it appeared to the company that the point of radiation was moving eastward from the zenith, when it occurred to the writer to mark its place, accurately, among the fixed stars.

The point was then seen to be in the constellation Leo, within the bend of the *sickle*, a little to the westward of Gamma Leonis, and not far from Regulus. During the hour following, the radiant point remained stationary in the same part of Leo. although the constellation in the mean time, by the diurnal revolution, moved westward to the meridian 15 degrees. By referring to a celestial globe, it will be seen, that this point has a right ascension of 150 degrees, and a declination of about 20 degrees. Consequently it was 20 degrees 18 minutes south of our zenith.

The weather had sustained a recent change. On the evening of the 11th, a very copious southerly rain fell, and on the 12th, a high westerly wind prevailed, by gusts. Last evening the sky was very serene; a few falling stars were observed, but not so numerous as to excite particular attention."

These notices will be sufficient to indicate the varieties of aspect which this great meteoric phenomenon put on, in different parts of our country; over the whole of which it appears to have been observed. To what distance it was seen west of Missouri, south of New Orleans, north of Canada, or to the east, over the Atlantic Ocean, we are as yet unable to say; but the commander of the ship Phœnix, reports it to have been observed by him and his crew, in long. 63 deg. 22 min. W., and 32 deg. 24 min. north: that is, about 12 deg. east of Charleston.

Several observers in this city, and elsewhere, had the impression, that some of these meteors actually fell to the earth; but none of them heard the sound of any thing solid striking upon the surface of the ground, nor have any fragments been found; so that, on the whole, we may conclude they were not aerolites, although those meteors, when appearing at night, have generally been luminous. It can scarcely, we think, be doubted, but that the stony meteors which occasionally fall, are distinct from the ordinary shooting-stars, to which those of the 13th of November would seem to have been analogous.

Several observers heard a faint and distant sound in the air, which was in some cases dull, in others, rushing or hissing; but the greater number of explosions, as they have been incorrectly called, were only dispersions, and attended with no noise whatever. Nor does there appear to have been any odour observed, even by those nearest the spot where the meteors were supposed to fall. To us it is not improbable, that none of them approached the surface of the earth, or even came into the lower regions of the atmosphere. The area of country over which they were observed, is already known to be so extensive, as to indicate, either that they were at a very great height, or that they occupied a vast horizontal space in the heavens.

For half a century, the nature of the shooting or falling-star, has been a subject of speculation with the scientific. The perpetual recurrence of that solitary meteor, every night, over the whole surface of the globe, would seem to show, that it does not depend on an occasional cause reproduced at distant periods; but a power constantly present in the upper regions, where that phenomenon is generated. Some philosophers have satisfied themselves with the opinion, that this cause is electricity; but until it can be shown how the electric fluid exerts itself to produce this appearance, the opinion can be regarded as nothing more than a hypothesis. Others have assumed the combustion of phosphoretted hydrogen, accumulated far over our heads, and taking fire spontaneously, on descending into denser strata of air; but whence was this gas derived, and what would cause it to fall through the atmosphere with the velocity which these meteors usually exhibit? On the whole, it must be admitted, that many facts are still wanting; and that we are nearly ignorant of the cause, not only of the late shower of falling-stars, but of the common and solitary meteor bearing that name.

As physicians, it is important to know, whether the late shower has been followed by any new disease, or any modification of the old. In reference to this city, nothing of the kind has occurred. We never had a healthier month than November, and December has presented nothing uncommon, either in the variety or force of our diseases. A similar remark would not be exactly applicable to our weather, for October and November were unusually cold; December, however, has had a compensating mildness, and these anticipations of winter, are by no means unprecedented. In respect to clouds, rain, winds, &c., the autumn and winter, thus far, have not been in any wise remarkable.

The great exhibition of shooting-stars, of which we have here embodied some notices, is not the first that has been presented to the admiration of the new world. On the morning of the 12th of November, 1799, just 34 years previously, there occurred a similar phenomenon. The late Professor Ellicott, of the Military Academy, employed by the government to run the boundary line between the United States and the Spanish possessions, when on board a vessel off the coast of Florida, observed a similar phenomenon, and as his name is frequently mentioned at the present time, we shall quote from his journal, now an exceedingly rare book, the whole of his observations:

November 12, 1799.—"About two o'clock in the morning, I was called up to see the shooting of the stars; (as it is vulgarly termed). The phenomenon was grand and awful; the heavens appeared as if illuminated with sky-rockets, flying in an infinity of directions, and I was in constant expectation of some of them falling on the vessel.—They continued until put out by the light of the sun, after daybreak. This phenomenon extended over a large portion of the West India Islands, and was observed as far north as St. Mary's, where it appeared as brilliant as with us. During this singular appearance, the wind shifted from the south to the north, and the thermometer, which had been at 86 degrees for four days past, fell to 56 degrees."

The same phenomenon was observed by Humboldt and Bonpland, when on their travels in South America, and we shall extract from the abridgment of their narrative, the following notice of it:

"Thousands of fireballs and falling-stars succeeded each other during four hours, having a direction from north to south, and filling a space of the sky extending from the true east 30 degrees on either side. They rose above the horizon at E. N. E. and at E., described arcs of various sizes, and fell towards S., some attaining a height of 40 degrees, and all exceeding 25 or 30 degrees. No trace of clouds was to be seen, and a very slight easterly wind blew in the lower regions of the atmosphere. All the meteors left luminous traces from five to ten degrees in length, the phosphorescence of which lasted seven or eight seconds. The fireballs seemed to explode, but the largest disappeared without scintillation; and many of the falling stars had a very distinct nucleus, as large as the disk of Jupiter, from which sparks were emitted. The light occasioned by them was white,—an effect which must be attributed to the absence of vapors; stars of the first magnitude having, within the tropics, a much paler hue at their rising than in Europe.

As the inhabitants of Cumana leave their houses before four, to attend the first morning mass, most of them were witnesses of this phenomenon, which gradually ceased soon after, although some were still perceived a quarter of an hour before sunrise.

The day of the 12th November was exceedingly hot, and in the evening the reddish vapor reappeared in the horizon, and rose to the height of 140 degrees. This was the last time it was seen that year.

The researches of M. Chladni having directed the attention of the scientific world to fireballs and falling-stars at the period of Humboldt's departure from home, he did not fail to inquire, during his journey from Caraccas to the Rio Negro, whether the meteors of the 12th November had been seen. He found that they had been observed by various individuals in places very remote from each other; and on returning to Europe was astonished to find that they had been seen there also. The following is a brief account of the facts relating to these phenomena:—1st, The luminous meteors were seen in the E. and E. N. E. at 40 degrees of elevation, from 2 to 6 A. M., at Cumana, in lat. 10 deg. 27 min. 52 sec., long. 66 deg. 30 min.; at Porto Cabello, in lat. 10 deg. 6 min. 52 sec., long. 67 deg. 5 m.; and on the frontiers of Brazil, near the equator, in long. 70 deg. west.—2dly, The Count de Marbois observed them in French Guiana, lat. 40 deg. 56 min., long. 54 deg. 35 minutes.—3dly, Mr. Ellicot, astronomer to the United States, being in the Gulf of Florida on the 12th November, saw an immense number of meteors, some of which appeared to fall perpendicularly; and the same phenomenon was perceived on the American continent as far as lat. 30 deg. 42 minutes.—4thly, in Labrador, in lat. 56 deg. 55 min. and lat. 58 deg. 4 min.; in Greenland, in latitudes 61 deg. 5 min. and 64 deg. 14 min., the natives were frightened by the vast quantity of fireballs that fell during twilight, some of them of great size.—5thly, In Germany, Mr. Zeissing vicar of Itterstadt near Weimar, in lat. 50. deg. 59 min., long. 9 deg. 1 min. E., observed, between 6 and 7 in the morning of the 12th November, some falling-stars having a very white light. Soon after reddish streaks appeared in the S. and S. W.; and at dawn the south-western part of the sky was from time to time illuminated by white lightning running in serpentine lines along the horizon.

Calculating from these facts, it is manifest that the height of the meteors was at least 1419 miles; and as near Weimar they were seen in the S. and S. W., while at Cumana they were observed in the E. and N. E., we must conclude that they fell into the sea between Africa and South America, to the West of the Cape Verd Islands.

Without entering into the learned discussion which Humboldt submits to his readers, respecting the nature of these luminous bodies, we shall merely observe, that he found falling-stars more frequent in the equinoctial regions than in the temperate zone, and also that they occurred oftener over continents and near certain coasts than on the ocean. He states, that on the platform of the Andes, there was observed, upwards of forty years ago, a phenomenon similar to that related above as having occurred at Cumana. From the city of Quito an immense number of meteors was seen rising over the volcano of Cayambo, insomuch that the whole mountain was thought to be on fire. They continued more than an hour, and a religious procession was about to be commenced, when the true nature of the luminous appearance was discovered."

It would appear from the following republication, that about four years after the event just narrated, it was reproduced, over one spot if not more:

From the Richmond (Va.) Gazette of April 3, 1803.

SHOOTING-STARS.

This electrical phenomenon was observed on Wednesday morning last, at Richmond and its vicinity, in a manner that alarmed many, and astonished every person who beheld it. From oneu ntil three in the morning, those starry meteors seemed to fall from every point in the heavens, in such numbers as to resemble a shower of sky-rockets.—The inhabitants happened at the same hour to be called from their houses by the fire bell, which was rung on account of a fire that broke out in one of the rooms of the armory, but which was speedily extinguished. Every one, therefore, had an opportunity of witnessing a scene of nature, which never before was displayed in this part of the globe, and which, probably, will never appear again.

Several of those shooting meteors were accompanied with a train of fire that illuminated the sky for a considerable distance. One, in particular, appeared to fall from the zenith, of the apparent size of a ball eighteen inches diameter, that lighted for several seconds the whole hemisphere. During the continuance of this remarkable phenomenon, a hissing noise in the air was plainly heard, and several reports, resembling the discharge of a pistol. Had the city bell not been ringing, these reports would probably have seemed much louder. The sky was remarkably clear and serene, and the visible fixed stars numerous the whole night. We are anxious to know at what distance from Richmond this phenomenon has extended. It is hoped that persons who have remarked it in other places, will not neglect to inform the public of the particulars; as such information may add, in a great degree, to the knowledge of Meteorology.

Since writing the above, we have been informed, that several of the largest of these shooting meteors were observed to descend almost to the ground before they exploded. Indeed, many of those which we saw, appeared to approach within a few yards of the house tops, and then suddenly to vanish. Some persons, we are told, were so alarmed, that they imagined the fire in the armory was occasioned by one of these meteors, and in place of repairing to extinguish the earthly flames, they busied themselves in contriving to protect the roofs of their houses from the fire of heaven.

This circumstance of the shooting-stars descending within a short distance of the ground, is, however, a fact highly important to be known; as it has been generally supposed, that meteors only proceed in a horizontal direction, and never fly perpendicularly upwards.—Those which we particularly remarked, appeared to descend in an angle of sixty degrees with the horizon; but as the smaller ones were so numerous, and crossed each other in different directions, it was only possible to ascertain with any precision, the paths of the largest and most brilliant."

